# Autologous Micrografts from Scalp Tissue: Trichoscopic and Long-Term Clinical Evaluation in Male and Female Androgenetic Alopecia

**DOI:** 10.1155/2020/7397162

**Published:** 2020-01-27

**Authors:** Pietro Gentile, Maria Giovanna Scioli, Valerio Cervelli, Augusto Orlandi, Simone Garcovich

**Affiliations:** ^1^Surgical Science Department, Plastic and Reconstructive Surgery, Tor Vergata University, Rome 00173, Italy; ^2^Biomedicines and Prevention Department, Anatomy Pathologic, Tor Vergata University, Rome 00173, Italy; ^3^Institute of Dermatology, F. Policlinico Gemelli IRCSS, Università Cattolica Del Sacro Cuore, 00168 Rome, Italy

## Abstract

Tissue engineering in hair regrowth aims to develop innovative and not-invasive procedures to advance the hair regrowth. A placebo-controlled, randomized, evaluator-blinded, half-head group study to compare hair regrowth with micrografts containing human hair follicle mesenchymal stem cells (HF-MSCs) vs. placebo was reported. After 58 weeks, 27 patients displayed in the targeted area an increase of hair count and hair density, respectively, of 18.0 hairs per 0.65 cm^2^ and 23.3 hairs per cm^2^ compared with baseline, while the control area displayed a mean decrease of 1.1 hairs per 0.65 cm^2^ and 0.7 hairs per cm^2^ (control vs. treatment: *P* < 0.0001). After 26 months, 6 patients revealed dynamic hair loss and were retreated. More broad controlled examinations are required. HF-MSCs contained in micrografts may represent a safe and viable treatment alternative against hair loss.

## 1. Introduction

For therapeutic hair regrowth, the use of micrografts containing autologous human hair follicle mesenchymal stem cells (HF-MSCs) has not been adequately considered. Androgenetic alopecia (AGA) is a typical, chronic hair loss disorder, described by dynamic hair loss, experienced interestingly by 80% of white men and 40% of ladies [1–3]. Medications and treatments affirmed for AGA are minoxidil, finasteride, and hair transplant [2]. The effect of autologous platelet-rich plasma (A-PRP) has been exhibited [3, 4]. In AGA, the miniaturization of the follicles is determined by diminishment of anagen and with an improvement in the percentage of resting hair follicles (HFs), telogen, containing microscopic hairs in a hairless scalp [5]. Moreover, invading lymphocytes and mast cells have been seen around the miniaturizing follicle [6], detailed in the stem cell-rich lump zone [7].

In hair loss scalp, hair follicle stem cell numbers stay unaltered, though the number of more actively proliferating progenitor cells particularly diminishes [8]. This proposes going bald scalp either does not have an activator or has an inhibitor of hair follicle (HF) growth.

In a previous study [9], the authors showed the use of autologous micrografts, reporting mechanical detachment of human hair follicle stem cells (HFSCs) is not expanded by a slow centrifugation according to minimal manipulation rules.

Right now, the authors intend to clarify the clinical and trichoscopic effects of micrograft scalp infusion in people affected by AGA.

Additionally, patients' fulfillment and computerized trichogram examination have affirmed the quality of the outcomes.

### 1.1. Scope of the paper

The objective of the present work is to evaluate the hair regrowth obtained by micrograft injections. The authors report here the long-term clinical efficacy of micrograft injections and compare also the results obtained with placebo. This report would also provide a concise review of recent advances in this field.

## 2. Methods

### 2.1. Study Overview

The primary outcome for the placebo-controlled, randomized, evaluator-blinded, half-head group study was to compare long-term results in hair regrowth with composite micrografts enriched with HF-MSCs vs. placebo (saline solution). The secondary outcome was to confirm through histological evaluation the follicle quantity, safety, and feasibility in HF-MSC-treated skin biopsies. Evaluation of trichograms was performed by medical doctors blinded to the procedure. AGA diagnoses were established by performing detailed therapeutic history, clinical examination, blood test, urinalysis, and trichoscopic highlights.

The grade of AGA in the selected patients was estimated according to the Norwood–Hamilton (NH) and Ludwig (L) scales.

### 2.2. Patients

This investigation enlisted 27 patients, of whom 17 males showed AGA grades 2–5 as controlled by the NH scale and 10 females showed AGA grades 1-2 as dictated by the L scale. Fundamental exclusion criteria included immunosuppression and cancer, sepsis, and the utilization of pharmacological therapeutics targeting on AGA (finasteride, similar drugs, and/or antiandrogens) in the earlier year. Localized exclusion criteria included the utilization of topical medicines for AGA (lotions as minoxidil, prostaglandin analogs, retinoids, or corticosteroids) in the earlier year.

### 2.3. Micrograft Procedure

**Figure 1:**
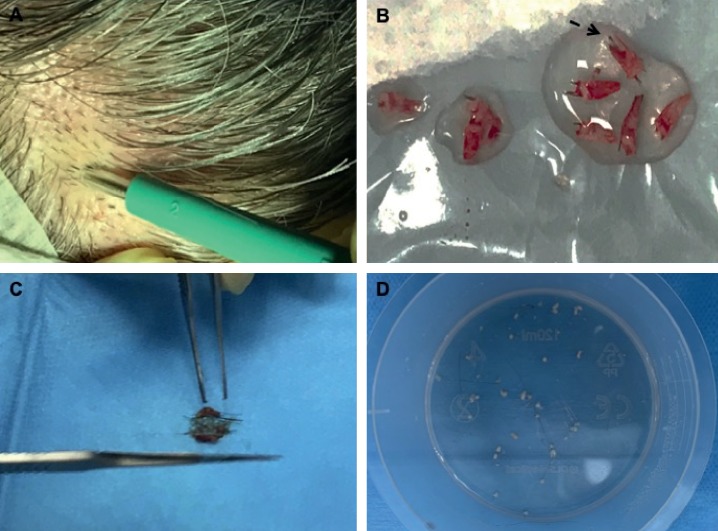
Micrograft's preparation with the “Gentile protocol” (first and second steps). (a) Harvesting of the scalp tissues with punch biopsy (2 mm diameter). (b) Storing scalp tissues in saline solution. (c) Cutting and splitting of the fragments into strips of 2.0 × 2.0 mm (group of 3 fragments each time) sterilely through a manual splitting performed by multiple incisions with scalpel number 11 in 1.2 mL of saline for each 3 fragments. (d) Splitted strips and obtained suspension; an average 20 ml for 50 fragments was disaggregated and fragmented.

**Figure 2:**
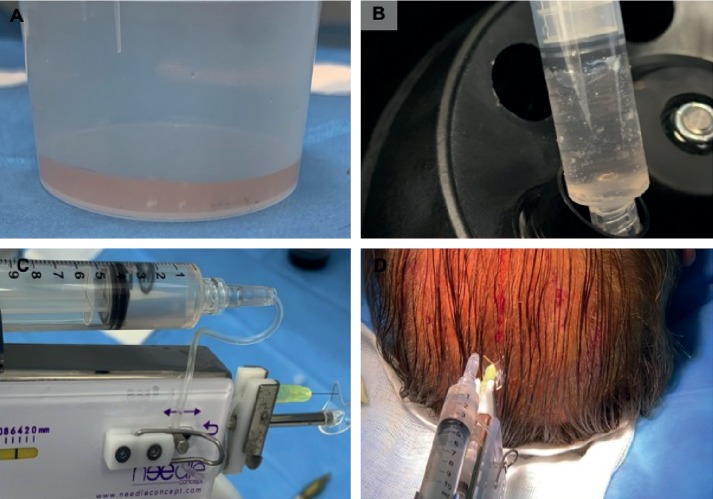
Micrograft's preparation with the “Gentile protocol” (third step). (a) 20 ml of suspension containing strips splitted. (b) Collection of the suspension obtained, in two 10 mL Luer-Lock syringes, and centrifugation for 3 minutes at 3000 RPM. (c) Two syringes containing 4.5 ml of the micrograft's suspension were positioned in a mesotherapy gun. (d) Mechanical and controlled infiltration, using 10 ml syringes in the selected area of the scalp through a mesotherapy gun.

Autologous micrografts of HFSCs were prepared using the “Gentile protocol” (Figures 1(a)–1(d) and 2(a)–2(d)), modifying and improving the procedure published previously [9]. In brief, this procedure represents an innovative clinical approach to obtain autologous micrografts through a mechanical fragmentation of different biological tissues (epidermis, dermal, fat tissue, hair, bulb area, and bulge area) and requires different steps of execution. The first step is harvesting of the scalp tissues (30–50 fragments depending on the size of the area to treat) with punch biopsy (2 mm diameter) (Figure 1(a)), storing in saline solution (Figure 1(b)), and cutting the fragments into strips of 2.0 × 2.0 mm (Figure 1(c)), resulting in collection and disaggregation of the strips (group of 3 fragments each time) sterilely through a manual splitting performed by multiple incisions with scalpel number 11 (Figure 1(c)) in 1.2 mL of saline (NaCl 0.9%) for each 3 fragments with the aim of sorting a cell group having a diameter of 80–120 *μ*m. The second step is collecting the suspension obtained; an average 20 ml for 50 fragments was disaggregated and fragmented (Figures 1(d) and 2(a)), in two 10 mL Luer-Lock syringes, and centrifuged for 3 minutes at 3000 RPM (Figure 2(b)); 1.5 mL of the supernatant was removed from each syringe, and 9 ml of the micrograft's suspension was obtained; two syringes containing 4.5 ml of the micrograft's suspension were positioned in a mesotherapy gun (Figure 2(c)). The third step is mechanical and controlled infiltration, using 10 ml syringes in the selected area of the scalp through a medical device, a mesotherapy gun, equipped with software that allows to schedule the depth of injection and the amount of infiltration per cm^2^, with the same angle of inclination (Figure 2(d)).

### 2.4. Protocol and Injection of the Micrograft's Suspension

The scalp was divided into six different anatomical areas (left frontal area, right frontal area, left parietal area, right parietal area, left vertex area, and right vertex area); anesthesia (local or potentially fundamental) was not performed. The micrograft's suspension interfollicular infusions (0.2 mL/cm^2^) were performed to the targeted area (TA) at 5 mm depth utilizing a mesotherapy medical device outfitted with a 10 mL Luer-Lock syringe (Supplemental Material) with a 30-gauge needle, in three sessions spaced 45 days apart. The micrograft's suspension infusions were conveyed to the frontal scalp, while placebo infusions (saline solution NaCl 0.9%) were infused in the parietal areas in patients with thinning confined to the frontal and parietal areas. In a similar manner, for thinning restricted to the parietal and vertex regions, micrografts' suspension was infused in the parietal area, and placebo was infused in the vertex area. Specifically, to better show the clinical impacts, we partitioned every area into two sides and performed the infusions in a chosen side (left or right) (Supplemental Material). Equivalent quantities of the autologous micrograft's suspension and placebo infusions were made.

### 2.5. Clinical Evaluation of Hair Growth

**Figure 3:**
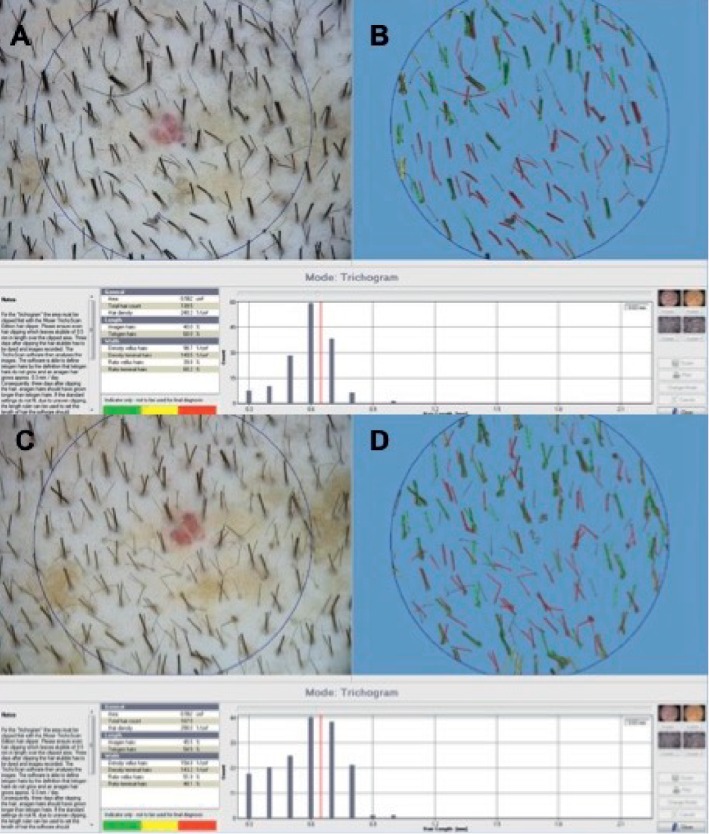
TrichoScan analysis performed by FotoFinder in a 41-year-old male with AGA who is nonsmoker; he is at stage 3 vertex balding according to the NH scale, as shown in Figure 4. (a, b) At T0, preoperative HC was 72.2 hairs per 0.65 cm^2^, HD was 97.5 hairs per cm^2^, and proportions of anagen and telogen hairs were 59.0% and 41.4%, respectively. (c, d) At T5, postoperative HC was 90.5 hairs per 0.65 cm^2^, HD was 122.8 hairs per cm^2^, and proportions of anagen and telogen hairs were 48.6% and 51.7%, respectively.

**Figure 4:**
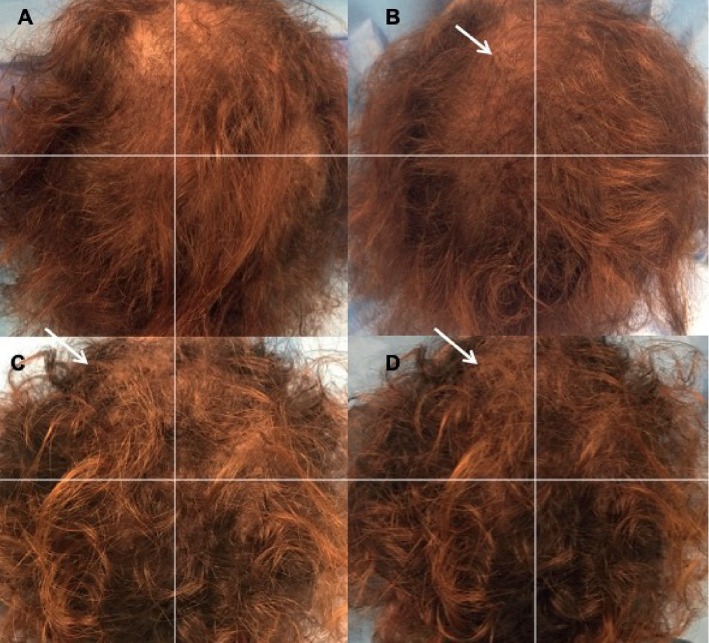
A 38-year-old female with AGA who is nonsmoker and classified to have stage 2 vertex balding according to the L scale. (a) Preoperative image at T0 with thinning localized to the frontal and parietal areas. (b) Postoperative image at T2 (9 Ws after the last treatment); the arrow indicates the left parietal area treated with two micrograft injections with the increase of HD vs. the right parietal area treated with placebo. (c) Postoperative image at T4 (23 Ws after the last treatment); the arrow indicates again the same area with the increase of HD. (d) Postoperative image at T5 (58 Ws after the last treatment); the arrow indicates the final result obtained.

**Figure 5:**
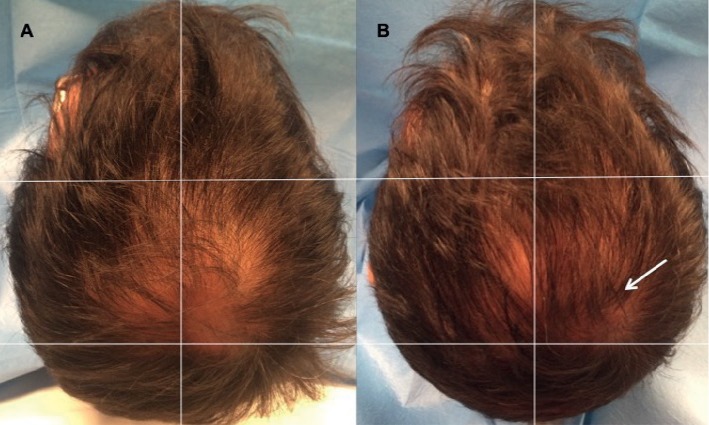
A 41-year-old male with AGA who is nonsmoker and classified to have stage 3 vertex balding according to the NH scale. (a) Preoperative image at T0 with thinning localized to the vertex, parietal, temporal, and frontal areas. (b) Postoperative situation of the scalp at T5-58 Ws after the last treatment with the arrow indicating improvement of HD in the right vertex area treated with two micrograft injections vs. baseline situation in the same area and vs. the left vertex area treated with placebo.

Assessment of hair growth (HG) was in different weeks (Ws) after the treatment, summarized into four phases: T0 (Figures 3(a) and 3(b)), before the first infusion (Figures 4(a) and 5(a)); T1-3 Ws and T2-9 Ws after the last treatment (Figure 4(b)); T3-16 Ws and T4-23 Ws after the last treatment (Figure 4(c)); and T5-58 Ws after the last treatment (Figures 3(c), 3(d), 4(d), and 5(b).

The impacts of micrografts' suspension and placebo medications on HG were evaluated using photography (same position, same contrast, and same light), the physician's and the patient's global evaluation scale, and standardized phototrichograms.

### 2.6. Trichoscopic Evaluation of Hair Growth

In all participants, two translational regions of thinning, one at the fringe of the treatment half and the other at a moment along the outskirt of the placebo half, have been marked with a semipermanent tattoo for the subsequent trichogram.

Phototrichograms (Figures 3(a)–3(d)) were performed in all TA using FotoFinder video epiluminescence systems in combination with the TrichoScan digital image analysis. TrichoScan was performed to evaluate hairs per 0.65 cm^2^ described as hair count (HC), hairs per cm^2^ described as hair density (HD), hair thickness (HT), anagen-to-telogen ratio, and vellus hair-to-terminal hair ratio. All hairs with a thickness >40 *μ*m are categorized as terminal hairs, while those with lesser diameter are categorized as vellus hairs.

### 2.7. Immunocytochemistry and Cytospin

Micrografts' suspension tests (*n* = 27) were fixed with 4% paraformaldehyde and characterized for mesenchymal CD44 [10] and epithelial CD200 [11] stem cell markers, according to previously reported studies [10, 11]. After cytospin, cells were analyzed by immunocytochemistry with specific primary antibodies (CD200 ab203887, 1 : 100; CD44 sc-9960, 1 : 10). Positive cells were counted in the total area (as percentage of positive cells/total cell number) under a light microscope at 400x magnification (Eclipse E600, Nikon, Japan), and microphotographs were captured by a DXM1200F digital camera (Nikon) using ACT-1 software (Nikon).

### 2.8. Statistical Analysis

HD and HC were expressed as mean plus or minus standard deviation (SD). HD and HC differences between the different time points were evaluated by one-way repeated-measures analysis of variance; post hoc analysis was performed using the Sidak test. All tests were 2-tailed, and *P* < 0.05 was considered statistically significant.

## 3. Results

### 3.1. Long-Term Clinical and Trichoscopic Results

The results obtained displayed an improvement in the mean HC at T5 after 58 weeks (58 weeks vs. 0 weeks) of 18.0 hairs in the TA (Figures 3(c) and 3(d)) compared with baseline (Figures 3(a) and 3(b)), while the control area (CA) displayed a mean decrease of 1.1 hairs (control vs. treatment: *P* < 0.0001). Accordingly, a mean increase in the total HD of 23.3 hairs per cm^2^ (Figures 3(c) and 3(d)) compared with baseline (Figures 3(a), 3(b), 4(a), and 5(a)) was observed at T5 (Figures 4(d) and 5(b)), and the CA displayed a mean decrease of 0.7 hairs per cm^2^ (control vs. treatment: *P* < 0.0001). There were no statistically significant differences in vellus HD between the TA and the CA at T5. After 26 months, 6 patients detailed dynamic hair loss. Those six patients were retreated.

### 3.2. Immunophenotypic Identification and Cell Counting

Twenty-seven samples of the micrograft's suspension were cultured and subsequently characterized by cytospin and immunocytochemistry to identify the HFSCs. The authors considered the cellular population containing HF-MSCs and human hair follicle epithelial stem cells (HF-ESCs) as HFSCs.

**Figure 6:**
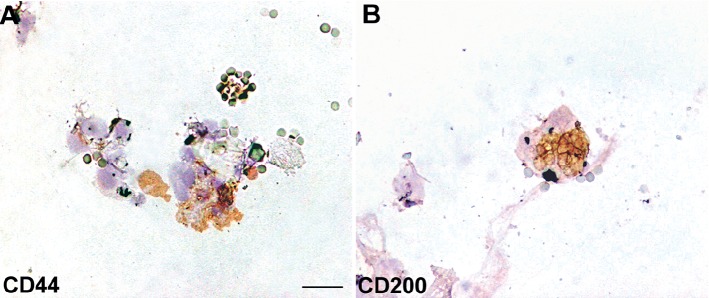
Immunophenotypic analysis of HFSCs in human scalp biopsy. Immunocytochemistry for CD44 and CD200 stem cell markers. (a) HF-MSCs. (b) HF-ESCs. Scale bar = 25 *μ*m.

From cell counting, each scalp tissue suspension contained a mean of about 4123.7 ± 581.8 cells. In particular, the percentage of CD44^+^ HF-MSCs (from the dermal papilla) (Figure 6(a)) was about 4.6% ± 0.65%, whereas the percentage of CD200^+^ HF-ESCs (from the bulge area) (Figure 6(b)) was 2.4% ± 0.4%. The remaining cells were S100A4+ dermal fibroblasts (>87%) and epidermal cells (epithelial cells and melanocytes < 12%).

## 4. Discussion

Current medications for AGA with the approval of the US Food and Drug Administration (FDA) include minoxidil and finasteride.

Minoxidil (pyrimidine derivate) lotion 2% was the first drug with FDA approval for treatment of AGA in males (1988) and females (1991) [12, 13]. Minoxidil as 5% lotion was approved in 1997 for AGA in males and as 5% foam in 2006 [12, 13]. Minoxidil extends the anagen period and increases HF diameter through activation of prostaglandin-endoperoxide synthase-1, which increases the level of prostaglandin E2 [12]. Minoxidil improves the survival of dermal papilla cells (DPCs) by activating ERK and Akt and by increasing the Bcl-2/Bax ratio [13].

Finasteride is a type II 5-alpha-reductase inhibitor which decreases dihydrotestosterone (DHT) by about 65% in serum, prostate, and scalp. It was registered in Europe in 1992 for treatment of benign prostatic hyperplasia [14, 15]. The drug became registered in the United States of America (1993) and Europe (1994) for AGA therapy (mild to moderate) in male patients [14, 15]. Oral finasteride also prolongs the hairs' anagen phase, with the gradual improvement of hair thickness [14]. Finasteride reduces the thinning, increasing the expression of caspase and apoptosis inhibitors, stimulating the anagen phase resulting in HG [15, 16].

Alternative treatments, based on autologous sources and minimal invasive approach, are represented by A-PRP and adult stem cells (SCs) as adipose-derived mesenchymal stem cells (AD-MSCs) and HFSCs [17]. A more invasive surgical approach was limited to hair transplant indicated only for patients affected by aggressive conditions of AGA and hair loss [17].

The clinical effectiveness of A-PRP as AGA therapy was reported in recent reports [3, 4, 17]. In this case, HG would be stimulated by antiapoptotic effects of A-PRP through the activation of the Bcl-2 protein (antiapoptotic regulator) and Akt signaling, prolonging the survival of DPCs during the hair cycle [3, 4, 17]. The upregulation of fibroblast growth factor-7 (FGF-7)/*β*-catenin signaling pathways with A-PRP was suggested to stimulate HG by inducing HFSC differentiation as well as prolonging the anagen phase of the HG cycle [3, 4, 17]. It also improved the perifollicular vascular plexus through the increase of vascular endothelial growth factor (VEGF) and platelet-derived growth factor (PDGF) levels, which have the angiogenic potential [3, 4, 17].

Adil and Godwin [18] reported selecting trials, based on the US Preventive Services Task Force quality assessment process, conducted separately for 5 groups of studies, in which the authors analyzed low-level laser light therapy, 5% minoxidil, 2% minoxidil, and 1 mg finasteride in male patients and 2% minoxidil in females. All the procedures were more effective compared to placebo (*P* < 0.00001), in the 5 meta-analyses.

To better explain the clinical results obtained by the use of minoxidil, finasteride, A-PRP, and HFSCs, it is necessary to report the most recent outcomes in hair density and hair count obtained for these treatments. In detail, in a recent study of Gentile et al. [19], hair density improvement for A-PRP 23 weeks after the third infiltration (one infiltration was performed three times for 30 days each) was 28 ± 2% hairs/cm^2^ compared with placebo (saline solution). In the same study, hair density improvement for HFSC treatment 23 weeks after the second infiltration (one infiltration was performed every 60 days for two times) was 29 ± 5% hairs/cm^2^ compared with placebo (saline solution) [19]. In a study of Van Nestle et al. [20], 212 males affected by AGA were selected in a randomized model to receive 1 mg finasteride daily (study group) or placebo (control group) for 48 Ws. At 48 Ws, the study group had a net improvement compared with the control group in total and anagen HCs of 17.3 ± 2.5 hairs (8.3% ± 1.4%) and 27.0 ± 2.9 hairs (26% ± 3.1%), respectively (*P* < 0.001).

In a recent study of Bao et al. [21], regarding the use of 5% minoxidil, the increase in HD 24 Ws after the treatment was 18.8 hairs/cm^2^ in the group of patients treated with topical application and 38.3 hairs/cm^2^ in the group of patients treated with electrodynamic microneedling with topical 5% minoxidil [21].

Regarding the effectiveness/expenses balance, it is possible to affirm that the drugs discussed could be effective in AGA patients but cause a dependence promoted by the need to take finasteride daily or apply minoxidil topically for 12–24 months according to the analyzed studies [20, 21].

On the contrary, the use of autologous therapies can free the patient from the daily routine, but the greater invasiveness of the procedures can lead the patient to be less compliant.

Adult SCs can be harvested, prevalently, from two tissues, represented by fat tissue (FT) and scalp tissue (ST). The FT contains a great number of mesenchymal stem cells (MSCs) with multilineage separation potential. The FT could be collected using a minimally aggressive procedure as a gentle liposuction, and it must be identified as a substitute to the bone marrow (BM) for surgical applications because it is gifted on high frequency of AD-MSCs [17].

AD-MSCs and stromal vascular fraction cells (SVFs) are vital for the ignition of epidermal SCs in the scalp, which are activated by the release of growth factors (GFs). The VEGF drives HG and the extension of the HFs measured by activation of angiogenesis. The PDGF prompts the anagen stage, and insulin-like growth factor-1 (IGF-1) controls the HG cycle [17]. Another action is the implementation of angiogenesis and improvement of the blood supply to DPCs. In addition, they have immunomodulatory and immunosuppressive activity through the release of leukemia-inhibiting factor (LIF), kynurenine, and prostaglandin E2 (PGE2) [17]. The paracrine activity of AD-MSCs and SVFs is based on TB4, EGR-1, SDF-1, and MCP-1 with implication of human HFs [17]. TB4 allows for the activation of SCs in HFs, increasing their relocation into the follicle and splitting. SDF-1 improves the cell tropism toward the follicle and increases the angiogenesis, through an activation improvement of EGR-1 [17]. MCP-1, despite having inflammatory capacity, has demonstrated a tissue regenerative effect [17].

It is likely that the anti-inflammatory and immunomodulatory properties of PRP or dermal and progenitor stem cells may favor hair regrowth [22–24] since AGA, as known, is characterized by an inflammatory infiltrate, responsible for the inflammatory cytokine release [22].

Stoll et al. [25] suggested in a preclinical model that trauma of the skin produced by microneedling injections would induce long-term hair regrowth. In this study, five Ws after microneedling injections, hair regrowth started, followed by a hyperpigmentation reduction. 12 Ws later, a 90% improvement in coat coverage was reported at previously thinning areas. 12 months later, coat conditions remained stable [25].

As reported by Fertig et al. [26], the microneedling injection is not an aggressive treatment based on a simple procedure where small needles are rolled over the epidermis, stimulating collagen production, angiogenesis, and growth factor release. The microneedling procedure has been tested in a great range of dermatologic pathologies as alopecia areata and AGA [26].

For ST, in previous research conducted by Gentile et al. [9], a procedure was tuned to collect HFSCs with minimal manipulation based on the centrifugation of the scalp's fragments obtained by punch biopsy, without cellular extension or culture. In this procedure, we succeeded in cell counting and identifying CD44+ HF-MSCs and CD200+ HF-ESCs, according to previously reported studies [10, 11].

In patients affected by AGA with scalp presenting hair loss, the HFSC numbers were reported to stay invariant [8].

However, the reconstitution of a completely sorted-out and utilitarian HF from separated cells seeded in culture is a test as yet pending in regenerative medicine [27].

HFs contain a niche for grown-up stem cells represented by the lump, containing epithelial and melanocytic stem cells [28].

SCs in the hair lump, a differentiated structure inside the lower permanent portion of HFs, can create the interfollicular epidermis, sebaceous glands, and HF framework [29, 30].

The lump ESCs can reconstitute in a simulated *in vivo* framework similarly to a new HF [31, 32].

Yu et al. [28] demonstrated that human HFs contain a stem cell populace that may be separated into the smooth muscle cell, neuron, and melanocyte heredities in the induction medium. Their information demonstrates that Oct4-positive cells are available in the human epidermis, and the majority of them are situated in the HFs *in vivo*. Oct4 has a place in the family of POU-domain transcription factors that are communicated in pluripotent cells of the developing embryo and mediate pluripotency [33]. Each mature HF is a regenerating framework, which physiologically experiences cycles of growth (anagen), relapse (catagen), and rest (telogen) at various times in a grown-up's life [34]. In catagen, HFSCs are kept up in the lump. At this point, the resting follicle reenters anagen (regeneration) when legitimate molecular signals are given. Amid the late telogen to the early anagen phase, signals from the dermal papilla (DP) promote the hair germ and switch off lump stem cells to wind up activated [35].

Numerous paracrine factors are engaged with this crosstalk at various hair cycle stages, and some signaling pathways have been implicated [36–38]. In anagen, SCs in the lump offer ascent to hair germs; at this point, the transient increasing cells in the grid of the new follicle proliferate quickly to frame another hair filament [39].

At this point, it appears necessary to better know in which stage it is necessary to act. Regeneration of HFs was likewise seen in people [40] when dermal sheath tissue was utilized, which was adequate to regenerate additionally the DP structure. After implantation, the whisker DP was equipped for promoting HF regeneration holding the data to decide the hair fiber type and follicle size [41]. Grafting of dermal-inductive tissue was restricted by the way that it was impractical to produce more HFs than the one obtained from the donor tissues. To defeat this constraint, diverse methodologies and exploratory models utilizing freshly or cultured isolated cells from both dermal and dermal/epidermal origin were tried. The vast majority of them included neonatal and embryonic murine cells.

Balañá et al. [27] in a preclinical model created a epidermal-dermal skin substitute by seeding cultured human-derived HF-ESCs and DPCs, in an acellular dermal grid. This product was grafted into a wound produced on bare mice skin, and fourteen days later, in the treated area, histological structures reminiscent of a great range of phases of embryonic HF improvement were identified, demonstrating concentric cellular layers of human origin and expressing k6hf, keratin in epithelial cells of the companion layer. The results obtained suggested that both epithelial and dermal cultured cells from the grown-up human scalp in a dermal scaffold could create *in vivo* structures that reiterate embryonic hair improvement.

Kalabusheva et al. [42] aimed to build up a simulated HF germ, through combining postnatal human DPCs and skin epidermal keratinocytes (KCs) in a hanging drop culture. Blended HF germ-like structures showed the start of the epithelial-mesenchymal collaboration, including WNT pathway enactment and expression of follicular markers. The impact of DP cell niche components including dissolvable components and extracellular matrix (ECM) molecules was examined during the time spent on the organoid assembling and growth. The outcomes obtained showed that soluble components had little impact on HF germ generation and Ki67+ cell score inside the organoids although BMP6 and VD3 kept up effectively the DP character in the monolayer culture. Talavera-Adame et al. [43] revealed the biomolecular pathway involved in cellular therapy.

Specifically, it has been additionally demonstrated that Wnt/*β*-catenin signaling is necessary for the growth and upkeep of DPCs [44, 45]. The increment of Wnt signaling in DPCs is one of the principal factors that enhance HG [44].

Festa et al. [46] detailed that adipocyte progenitor cells bolster the SC niche and help conduct the complex HG cycle. This approach aimed to regenerate HFs which is fascinating and raises the likelihood that one can conduct or reestablish the hair cycle in the thinning by stimulating the niche with autologous fat improved with stromal cells.

Along these lines, Perez-Meza et al. [47] detailed the safety, tolerability, and in patients with hereditary thinning treated with subcutaneous scalp infusion of advanced FT. The discoveries propose that scalp stem cell-enriched fat grafting may represent a promising elective way for treating hair loss in people.

Fukuoka and Suga [48] reported a mean increment of 29 ± 4.1 hairs in male patients and 15.6 ± 4.2 hairs in females treated with fat-derived stem cell-conditioned medium infusion.

## Data Availability

All data generated and/or analyzed during this study are included in this article and its additional files.

## Abbreviations

HF-MSCs: Human hair follicle mesenchymal stem cells
HFSCs: Human hair follicle stem cells
HF-ESCs: Human hair follicle epithelial stem cells
AGA: Androgenetic alopecia
A-PRP: Autologous platelet-rich plasma
SPSS: Statistical Package for the Social Sciences
DPCs: Dermal papilla cells
DP: Dermal papilla (DP)
KCs: Keratinocytes
ECM: Extracellular matrix
CD: Cluster of differentiation
NaCl: Saline solution
HFs: Hair follicles
SVFs: Stromal vascular fraction cells
AD-MSCs: Adipose-derived mesenchymal stem cells
SCs: Stem cells
HG: Hair growth
GFs: Growth factors
VEGF: Vascular endothelial growth factor
PDGF: Platelet-derived growth factor
IGF-1: Insulin-like growth factor-1
FGF-7: Fibroblast growth factor-7
ST: Scalp tissue
FT: Fat tissue
NH: Norwood–Hamilton
L: Ludwig
TA: Targeted area
HD: Hair density
HC: Hair count.

## Supplementary Materials

Supplementary Materials Micrograft's injection procedure with a mesotherapy gun: fast, mechanical, and controlled injection in the targeted area affected by AGA.
